# Associations between spontaneous electroencephalogram oscillations and oxygen saturation across normobaric and hypobaric hypoxia

**DOI:** 10.1002/hbm.26214

**Published:** 2023-01-30

**Authors:** Evan A. Hutcheon, Vasily A. Vakorin, Adonay Nunes, Urs Ribary, Sherri Ferguson, Victoria E. Claydon, Sam M. Doesburg

**Affiliations:** ^1^ Department of Biomedical Physiology and Kinesiology Simon Fraser University Burnaby British Columbia Canada; ^2^ Harvard Medical School Harvard University Boston Massachusetts USA; ^3^ Department of Psychology Simon Fraser University Burnaby British Columbia Canada; ^4^ Environmental Physiology and Medicine Unit Simon Fraser University Burnaby British Columbia Canada

**Keywords:** blood oxygen, EEG, EEG oscillations, entropy, hypobaric hypoxia, normbaric hypoxia, oxygen desaturation, resting state, spectral power

## Abstract

High‐altitude indoctrination (HAI) trains individuals to recognize symptoms of hypoxia by simulating high‐altitude conditions using normobaric (NH) or hypobaric (HH) hypoxia. Previous studies suggest that despite equivalent inspired oxygen levels, physiological differences could exist between these conditions. In particular, differences in neurophysiological responses to these conditions are not clear. Our study aimed to investigate correlations between oxygen saturation (SpO_2_) and neural responses in NH and HH. We recorded 5‐min of resting‐state eyes‐open electroencephalogram (EEG) and SpO_2_ during control, NH, and HH conditions from 13 participants. We applied a multivariate framework to characterize correlations between SpO_2_ and EEG measures (spectral power and multiscale entropy [MSE]), within each participant and at the group level. Participants were desaturating during the first 150 s of NH versus steadily desaturated in HH. We considered the entire time interval, first and second half intervals, separately. All the conditions were characterized by statistically significant participant‐specific patterns of EEG–SpO_2_ correlations. However, at the group level, the desaturation period expressed a robust pattern of these correlations across frequencies and brain locations. Specifically, the first 150 s of NH during desaturation differed significantly from the other conditions with negative absolute alpha power–SpO_2_ correlations and positive MSE–SpO_2_ correlations. Once steadily desaturated, NH and HH had no significant differences in EEG–SpO_2_ correlations. Our findings indicate that the desaturating phase of hypoxia is a critical period in HAI courses, which would require developing strategies for mitigating the hypoxic stimulus in a real‐world situation.

## Graphical abstract

It is still unknown whether there are differences in neurophysiological responses to normobaric and hypobaric hypoxia, as normobaric hypoxia is often used as a proxy for hypobaric hypoxia during research and hypoxia awareness courses. We collected 5‐min of eyes‐open resting‐state data with an electroencephalogram (EEG) and oxygen saturation (SpO_2_) monitor from 13 participants during normobaric hypoxia, hypobaric hypoxia, and normobaric normoxia. We found that there is less variability in the spatiotemporal patterns of EEG–SpO_2_ correlations during the desaturation phase of normabric hypoxia when compared to a stable desaturated phase.

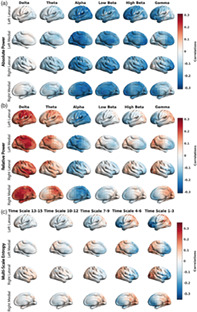

## 1 INTRODUCTION

Hypoxia is a serious concern for pilots, in whom the neurocognitive effects of hypoxia can have devastating consequences. Accordingly, high‐altitude indoctrination (HAI) programs are a requirement for military aviation to ensure pilots recognize the symptoms of hypoxia (Johnston et al., 2012; Shaw et al., 2021). Currently, HAI programs are performed with two different forms of hypoxia, either hypobaric hypoxia (HH), normobaric hypoxia (NH), or a mixture of both (depending on the country or organization). It is not known whether these different approaches equally recapitulate the relevant neurophysiological responses.

Alongside HAI, hypoxia research has a wide range of other applications: investigating the pathogenesis of illness characterized or complicated by hypoxia (Grocott et al., 2007); examining responses during acute high‐altitude exposure (Grant, 2002); enhancing training methods for performance athletes (Millet et al., 2013); working in a hypoxic environment for fire protection (Angerer & Nowak, 2003); and hypoxia risk in technical and occupational diving (Mitchell et al., 2019).

Hypoxia can be studied in a laboratory setting by either inducing HH (decreasing barometric pressure with an associated reduction in inspired oxygen) or NH (decreasing the fraction of inspired oxygen through increases in the partial pressure of nitrogen, without changing barometric pressure). It has long been assumed that a reduction in the inspired partial pressure of oxygen (PiO_2_) is the root cause of the hypoxia symptoms, and any combinations of barometric pressure with the inspired fraction of oxygen that produce equivalent PiO_2_ will have the same physiological effects (Conkin, 2016; Conkin & Wessel, 2008). A critique of using NH as an experimental model for hypoxia is that this oversimplifies the issue and ignores the potential different roles of nitrogen in the two scenarios (e.g., nitrogen enters tissues during NH and exits the tissues in HH) (Conkin, 2016).

The uncertainty in this area reflects that the available literature is conflicting. Standardization of study methods is an issue, with sample sizes, and confounding variables such as concurrent changes in humidity, temperature, accompanying cardiorespiratory responses, nitrogen, and end tidal carbon dioxide levels make comparing studies difficult. Oxygen saturation (SpO_2_) was found to be lower in HH than NH for exposures less than an hour, but this difference was lost with longer exposures (Coppel et al., 2015; Savourey et al., 2003; Self et al., 2011). Minute ventilation, alveolar ventilation (Basualto‐Alarcón et al., 2012; Coppel et al., 2015; Faiss et al., 2013; Loeppky et al., 1997; Savourey et al., 2003; Tucker et al., 1983), and subsequent tidal volume were decreased more in HH when compared to NH (Basualto‐Alarcón et al., 2012; Coppel et al., 2015; Faiss et al., 2013; Savourey et al., 2003, 2007; Tucker et al., 1983); however, end tidal partial pressure of carbon dioxide levels was similar in HH and NH (Coppel et al., 2015; Savourey et al., 2003, 2007; Tucker et al., 1983). It is unclear whether these differences influence how the brain responds to HH or NH.

If there are physiological differences between NH and HH, then neurophysiological differences in the responses to these conditions may also exist, which could lead to different hypoxia‐related cognitive impairments. It may be that cognitive impairment is more severe in HH as SpO_2_ is lower in HH than NH. This is supported by reports that decreased SpO_2_ is correlated with decreased executive function (Ochi et al., 2018) and that changes in arterial oxygen partial pressure have a greater impact on cognitive impairment than the barometric pressure change (Aebi et al., 2020; McMorris et al., 2017). In contrast, working memory impairment was noted to be similar between NH and HH (Malle et al., 2013, 2016). The limited data available and different experimental paradigms employed make it difficult to ascertain whether cognitive differences exist between NH and HH.

The temporal precision, relative low cost, and mobility of electroencephalogram (EEG) brain imaging make it an excellent neurophysiological method for studying hypoxia, particularly in the context of a hypobaric chamber or in remote and difficult locations (i.e., mountainous areas). Hypoxia has been found to induce changes in EEG signal power at various frequencies; however, methodological differences between studies make direct comparisons difficult. Interestingly, the impact of hypoxia on the alpha frequency band (8–12 Hz) depends on the participant's eye's being open or closed (Schellart & Reits, 2001), with increases in signal power during hypoxia in eyes‐open resting‐state and decreases in signal power in eyes‐closed resting‐state (Kraaier et al., 1988; Ozaki et al., 1995; Papadelis et al., 2007; Schellart & Reits, 2001). These responses are opposite to alpha responses to opening and closing one's eyes in normoxia (Barry et al., 2007). Hypoxia was also found to increase delta (Kraaier et al., 1988; Schellart & Reits, 2001), theta (Kraaier et al., 1988; Ozaki et al., 1995; Papadelis et al., 2007; Schellart & Reits, 2001), and beta power (Schellart & Reits, 2001; Schneider & Strüder, 2009), with some studies reporting increases in total power across the frequency spectrum (Papadelis et al., 2007; Schellart & Reits, 2001). Source space analysis of EEG during hypoxia also identified increases in low beta power (12.5–18 Hz) in the right superior frontal gyrus during a mild hypoxic stimulus (Schneider & Strüder, 2009). Conversely, theta, alpha, beta, and gamma power have been found to be significantly decreased during hypoxia (Rice et al., 2019). The contrasting findings in this study may be due to the use of a dry‐EEG system in which each sensor was contained within its own Faraday cage to reduce electrical noise, and a larger sample size than all previous studies. One limitation to comparing previous EEG studies on hypoxia is the differences in the level and duration of the hypoxic stimulus, whether NH or HH were employed, and advances in EEG technology and analysis methods that were not available in older studies.

In addition to conventional EEG measures such as spectral power, EEG is often characterized in terms of signal variability or signal complexity. Such characteristics provide a richer description of EEG rhythms, enabling consideration of the human brain as a highly complex system, with neuronal ensembles organized into functional networks (Wang et al., 2018). Various definitions of signal entropy have been used to describe EEG oscillations, which can be estimated using mathematical instruments such as information theory and nonlinear dynamics. There are multiple reports that these approaches provide additional benefits relative to traditional EEG measures, with an improved ability to differentiate between neurophysiological responses to different hypoxic conditions (Papadelis et al., 2007), enhanced detection of ischemia‐induced EEG changes (Bezerianos et al., 2003), and improved EEG classification of the neurophysiological impacts of Alzheimer's disease (Gomez et al., 2017; Wang et al., 2015).

Determining whether the neurophysiological impacts of NH and HH are distinct is important for research into hypoxic disease states and for HAI courses. If relevant differences do not exist, then HAI courses and hypoxia induction can be performed using NH, which is a safer, cheaper, and more accessible option than HH. Conversely, if a difference does exist between NH and HH then HAI courses and relevant hypoxia research should be performed using HH. To test the hypothesis that NH and HH are neurophysiologically distinct, we aimed to compare the neurophysiological response to NH and HH using EEG brain imaging. As previous studies found that SpO_2_ differs between NH and HH in acute hypoxia (Coppel et al., 2015; Savourey et al., 2003; Self et al., 2011), and decreased SpO_2_ has an association with decreased executive function (Ochi et al., 2018), we also aimed to investigate the relationships between SpO_2_ and neurophysiological responses to the two stimuli.

## 2 METHODS

### 2.1 Participants

The study was approved (#20160635) by the Simon Fraser University Research Ethics Board and all participants provided written informed consent. Participants were recruited from the university and local community. Participants were included if they had normal or corrected‐to‐normal vision. The exclusion criteria incorporated factors that might influence participant safety or comfort during altitude exposure such as pregnancy, smoking, respiratory disease, claustrophobia, nasal congestion, use of antihypertensive medication, anemia, psychiatric or neurological disorders, and any other medical conditions not compatible with altitude exposure. We also excluded participants with beards, in case this influenced the fit of the breathing mask, and individuals such a divers or pilots who may have developed physiological adaptations to hyperbaria and hypobaria that could influence their responses to the test. Participant demographic information for age, height, weight, and body mass index (BMI) were calculated and presented as mean ± standard deviation.

### 2.2 Experimental conditions

**FIGURE 1:**
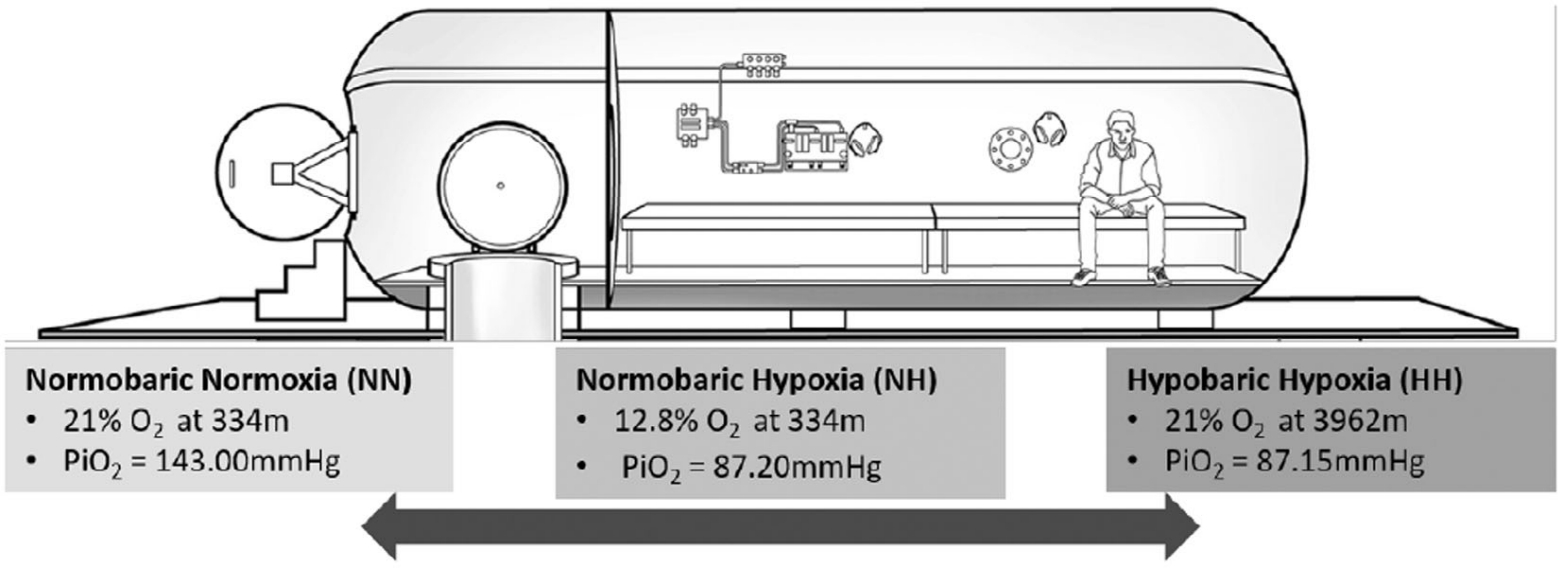
All three conditions were recorded within the hypobaric chamber. The chamber can be used for hyperbaric or hypobaric experiments, and for this study, it was in hypobaric mode. The NN and NH conditions were performed at 334 m.

Each participant was tested in three randomized conditions (Figure 1), each lasting for 5‐min. Tests were conducted in a single‐blind fashion. All conditions were conducted inside an altitude chamber located at 334 m above sea level. Recovery times between exposures were at least 48 h.

For the hypoxic conditions, we adopted an experimental design that maintained participant safety, while also having clear relevance to the stakeholder community. Canadian Aviation Regulations state that supplementary oxygen must be used for flights in unpressurized aircraft that are longer than 30 min at an altitude between 10,000 and 13,000 ft (3048–3962 m), with an upper limit for hypoxic exposure without supplemental oxygen or cabin pressurization equivalent to an altitude of 13,000 ft (3962 m). Accordingly, in the HH condition, participants experienced a decrease in pressure while breathing ambient air (21% oxygen, PiO_2_ = 87 mmHg) at a simulated altitude of 3962 m through a mask. The NH condition was performed with no change in pressure inside the hypobaric chamber, but with the participant breathing a hypoxic gas mixture calculated to elicit a PiO_2_ equivalent to the HH condition (12.8% oxygen, PiO_2_ = 87 mmHg) through a mask at 334 m. The NN condition was performed with no change in pressure inside the hypobaric chamber and with the participant breathing the ambient air (21% oxygen, PiO_2_ = 143 mmHg) at 334 m through a mask.

#### 2.2.1 Hypobaric chamber specifications

The chamber used in the current study was constructed according to PVHO‐One standards. The chamber is a triple‐lock, multiplace Class “A” Hypo/Hyperbaric complex that contains an entry lock, main lock, and wet pot. Note that the wet pot, located beneath the chamber, was not used in the present study and accordingly the hatch depicted that leads to it was closed for the present investigations. The chamber has a maximum working pressure capacity of 30 ATA (445 PSI) and a minimum working pressure of 0.01 ATA (0.15 PSI). The overall length, width, and height of the chamber are 7.3, 33.86, and 32.2 m, respectively. For safety, a chamber tender accompanied participants in the chamber for all exposures.

All gas mixtures were delivered inside the chamber, and participants wore modified masks (V2 mask Hans Rudolph Inc.) throughout the experiment for all three conditions.

### 2.3 Testing procedure

#### 2.3.1 Participant instrumentation

**FIGURE 2:**
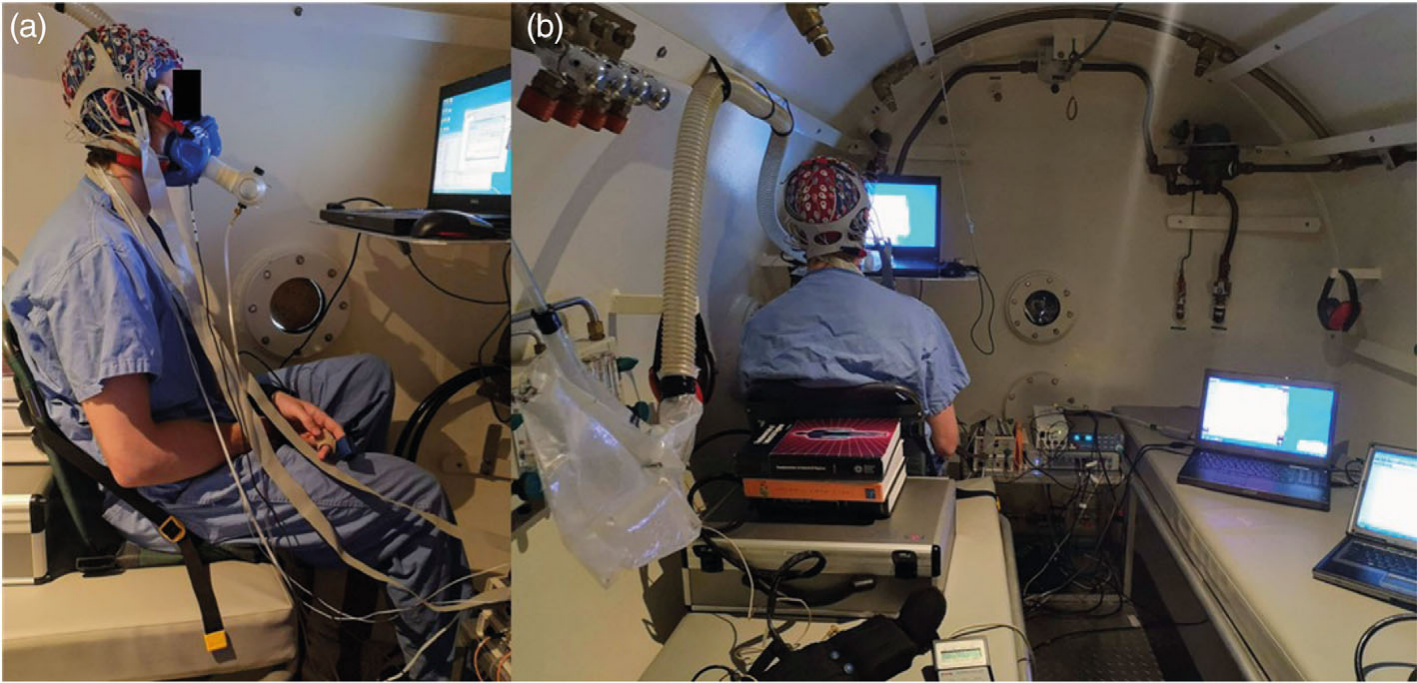
(a) View of participant set up from the side. The 64‐channel EEG cap is worn on the participant's head, with the mask straps going over the electrodes to maintain an adequate seal on the mask. An SpO_2_ monitor can be seen on the left index finger. A computer was positioned 60 cm in front of the participant's eyes that was used to provide a fixation cross for data collection during resting‐state conditions, and a subsequent cognitive function task. Note that the results of the cognitive function task are beyond the scope of the present study, which examines responses during eyes‐open resting‐state conditions. (b) Experimental set up seen from the back. The chamber tender was behind the participant and manually matched the flow rate of the gas (either hypoxic or ambient mixture) to the participant's breathing rate. The two laptops to the right of the participant were used to record the EEG and SpO_2_ data.

Participants were tested at either 9 a.m., 12:30 p.m., or 3:30 p.m. First, participants were fitted with an EEG and brought into the hypobaric chamber. Once in the hypobaric chamber, participants had an SpO_2_ monitor fitted to the left index finger (Nonin 7500 Pulse Oximeter), and the EEG and SpO_2_ were connected to the recording computer (Figure 2). Participants sat on a bench with their eyes 60 cm away from a computer screen. Once sitting, the modified mask was put on the participants; however, it was not yet connected to a gas mixture (participants breathed ambient chamber air through the mask).

#### 2.3.2 Blinded experimental setup

An experimental blind was employed so that participants could not differentiate between the experimental conditions. For the HH condition, the hypobaric chamber was depressurized to the equivalent of 1524 m followed by a return to ambient pressure. This is a required safety check to ensure that participants could successfully equalize the pressure in their ear canal. This quick depressurization to the equivalent of 1524 m was also performed for the NN and NH conditions for consistency and to ensure each participant was “blind” to the condition. For the HH condition, the chamber was depressurized to the equivalent of 3962 m with an average time of 3 min 20 s. For the NH and NN conditions, the altitude chamber made an equivalent depressurization noise, but no pressure change occurred in the chamber.

At the end of the experiment, participants in the HH condition were returned to ambient pressure, and participants in the NN and NH conditions were depressurized to 3048 m and subsequently repressurized to ambient pressure in the same amount of time it took to repressurize in the HH condition. This was done to create a similar sensation of pressure change in the ears in the NN and NH conditions, again to ensure each participant was “blind” to the condition. A mask was needed for the NH condition so that the participants could breathe a hypoxic gas mixture, so for consistency participants also wore a mask in the HH and NN conditions through which they breathed the ambient air (hypoxic ambient air in HH). Note that while great care was taken to ensure participants were blinded to the experimental condition, it was not possible to blind the chamber tender to the condition for safety reasons.

#### 2.3.3 Experimental protocol

Following depressurization to 3962 m (HH) or the experimental blind (NN, NH), the participant's masks were connected to either the hypoxic gas mixture (NH) or ambient chamber air (HH, NN). Participants performed the flight to 3962 m on ambient air. The chamber tender manually controlled the flow rate of the breathing gas to match the participant's breathing rate, making sure that the bag never collapsed on itself or was never overfilled (Figure 2). Data were then collected during a 5‐min eyes‐open resting‐state, in which participants were instructed to stare at a white fixation cross against a black background while EEG and SpO_2_ data were simultaneously recorded. Once finished, participants were repressurized to sea level (HH) or had a short depressurization as described above as part of the blind (NH, NN). If at any time the participant wanted to stop, or if their SpO_2_ dropped continuously below 80%, they were put on 100% oxygen and the experiment stopped.

### 2.4 EEG recording

EEG was recorded with an EEG‐Cap (BioSemi) with 64 Ag‐AgCL active‐electrodes (ActiveTwo) arranged in the international 10–20 system mounted on the participant's head. Electrodes were filled with Signa gel (Parker Laboratories, Inc.). The analog signal of the EEG was converted to digital signals using the ActiveTwo AD‐box (Biosemi) and recorded at a sample frequency of 512 Hz. Electrode offsets were kept below 50 mV for all electrodes.

### 2.5 EEG preprocessing data analysis

EEG recordings were preprocessed using the Fieldtrip toolbox (Oostenveld et al., 2011) in MATLAB 2019b. All statistical analyses were performed in MATLAB 2019b. First, we applied a 1–55 Hz band‐pass filter (note that the power line frequency in our case is 60 Hz), and then channels with excessive artifacts were visually identified and removed from further analysis. The EEG was referenced with the common average reference. Epochs containing significant muscle artifacts were visually identified and removed from further analysis. Independent component analysis (runica algorithm) was then used to identify and reject components containing eye blinks and saccades (Jung et al., 2000; Siew Cheok & Raveendran, 2008). The data were then split into 2 s epochs. Previously removed channels were then interpolated based on the EEG signal data from surrounding channels.

### 2.6 Source estimation

Separately, for each participant, neural activity (source dynamics) was reconstructed on a grid throughout brain space as the weighted output of the EEG channel array (a spatial filtering method). A standard boundary element method (BEM) volume conduction model (Oostenveld et al., 2003) was employed based on the “colin27” MRI (Holmes et al., 1998) utilizing a 3‐sphere (scalp, skull, brain) model to create a template head model (i.e., BEM forward model). The coordinates of the electrode positions were then applied to the scalp of the head model following the 10–20 system.

Based on the head model and resting‐state EEG data, a set of weighting coefficients (spatial filter) was defined on a 15 × 15 × 15 mm grid encompassing the whole brain using a linearly constrained minimum variance beamformer (Van Veen et al., 1997). The covariance matrix was regularized with a 5% diagonal loading. As a result, for each epoch, condition, and participant, we reconstructed neural activity at 590 locations throughout brain space.

### 2.7 EEG measures

Each EEG epoch was characterized with three EEG measures: absolute and relative spectral power, and multiscale entropy (MSE).

#### 2.7.1 Absolute power of EEG signals

For each subject, source, and EEG epoch, we estimated the absolute power of EEG dynamics with Welch's method at frequencies between 2 and 55 Hz with a 1 Hz step. As the result of this procedure, each EEG epoch, for each participant, was associated with a matrix (54 × 590) of absolute EEG power estimated at 54 frequency points and 590 brain locations.

#### 2.7.2 Relative power of EEG signals

For each epoch, EEG signals were rescaled (z‐normalized) such that each individual signal had the mean of zero and the SD of 1. Then, similar to the absolute EEG power, relative EEG power was estimated with the same Welch's method on the z‐normalized EEG. Accordingly, for each participant and each EEG epoch, there were 54 × 590 estimates of relative EEG power estimated at 54 frequency points and 590 source locations.

#### 2.7.3 MSE of EEG signals

In addition to EEG power, for the same EEG time series, we estimated MSE, a measure of signal variability, which can be linked to the signal power content (Courtiol et al., 2016; Humeau‐Heurtier, 2020; Vakorin & McIntosh, 2012). MSE is defined as sample entropy computed on different time scales. In turn, sample entropy was proposed as a refined version of approximate entropy to estimate the rate of information generated by a dynamic system (a neuronal ensemble) underlying the observed signal (EEG) (Richman & Moorman, 2000). Temporal scales for MSE are defined by down‐sampling the original time series at increasingly coarse time resolutions by averaging consecutive time points in the original signal. At scale 1, the coarse‐grained time series is the raw signal itself. At scale *n*, the coarse‐grained time series is formed by averaging n consecutive time points from the original signal. The downscaling procedure in MSE ultimately acts as a low‐pass filter with a decreasing cut‐off frequency. Subsequently, sample entropy is computed as a function of scale, similar to the estimation of signal power as a function of frequency. For each EEG source and epoch, we estimated sample entropy at time scales 1–15, with parameters (embedding dimension of 2 and characteristic length of 0.5) typical in EEG analysis (Mišić et al., 2015; Vakorin & McIntosh, 2012). A Pearson correlation was performed on the averaged sample entropy correlations calculated at time scale 2 with average absolute power correlations at 50 Hz and averaged relative power correlations at 50 Hz. This was done to investigate the link between signal power content and MSE.

### 2.8 Correlations between EEG measures and SpO_2_

For each participant, separately for each type of EEG measures (absolute power, relative power, and MSE), we correlated EEG measures and SpO_2_ across epochs. This was performed with behavioral partial least squares (PLS) (Krishnan et al., 2011). Using PLS, we considered all EEG features at once: all brain locations and all frequencies for EEG power or all time scales in MSE. Specifically, for each EEG epoch, we determined: (1) 31,860 estimates of absolute EEG power (54 frequencies × 590 EEG brain locations); (2) 31,860 estimates of relative EEG power; and (3) 8850 estimates of sample entropy (15 time scales × 590 brain locations). We performed the behavioral PLS on each participant for the full 5‐min of recording, the first 150 s of recording, and the last 150 s of recording.

Behavioral PLS is a commonly employed technique within the neuroimaging field (McIntosh et al., 1996). Specifically, using a behavioral PLS approach, we decomposed the covariances between EEG features and SpO_2_ into a set of latent variables (LVs), similar to principal component analysis. Each LV was associated with a scalar value representing an overall correlation between the EEG features and SpO_2_. The significance of this correlation was evaluated with a permutation test based on permuting EEG epochs. This is a “global” test, as it generates one *p* value for the overall correlation between EEG and SpO_2_ across all EEG features at once. Note that as behavioral PLS generates one *p* value for one overall correlation between SpO_2_ and all EEG features at once, such an approach alleviates the problem of multiple comparisons. In addition, a bootstrap test was applied to explore how this overall correlation was expressed across EEG features (in the feature space spanned by brain locations and frequencies or time scales). The bootstrap test is a “local” test, as it estimates the contribution of individual EEG features to the overall correlation. As a result, each unique combination of brain locations and frequencies (or time scales, as in MSE) was associated with a bootstrap ratio value, showing the robustness of the contribution of individual EEG features to the overall correlation. The bootstrap ratio value is equivalent to a z‐score. In our study, we use the bootstrap ratio values and z‐scores interchangeably. In addition to the z‐score, each EEG feature can be associated with a correlation value between an EEG measure and SpO_2_ across epochs. Both the permutation and bootstrap tests were based on 5000 random samples. All the feature‐specific correlations were organized as spatiotemporal maps across brain locations and frequencies (or time scales). Note that z‐scores can be positive or negative. If the z‐score is positive, then the sign of correlation between EEG and SpO_2_ for a given EEG feature is determined by the sign of the overall correlation value. In contrast, negative z‐scores indicate negative correlations between EEG measures and SpO_2_, if the overall correlation is positive.

### 2.9 Comparing overall correlations between EEG measures and SpO_2_ between conditions

We compared group averages for SpO_2_ and the correlations gained from the behavioral PLS. For each participant, the behavioral PLS analysis returned one value of overall correlation between EEG measures and SpO_2_. Nine separate nonparametric two tailed paired Wilcoxon signed‐rank tests were performed on the individual correlations averaged across conditions to look for condition differences within a specific brain feature (e.g., NH vs. NN for MSE‐SpO_2_ correlations). Similarly, three separate two tailed students paired *t*‐tests were performed to examine group differences in interval‐averaged SpO_2_ between conditions (i.e., NN vs. NH, NH vs. HH, and NN vs. HH). A Shapiro–Wilk test was performed on the SpO_2_ data to test for normality, and the data were normally distributed. The mean ± standard deviation were also calculated and reported for each condition. A statistical threshold of *p* = .05 was used for both *t*‐tests and Wilcoxon signed‐rank tests and both tests were corrected against multiple comparisons by using false discovery rate (FDR).

### 2.10 Differences in the spatiotemporal maps of correlations across conditions

We also tested how the spatiotemporal maps of correlations between EEG and SpO_2_ from single‐subject analysis, as described in the previous section, varied across conditions at the group level. We applied PLS analyses; separately for absolute power, relative power, and MSE and separately for three time intervals. More specifically, we applied mean‐centered PLS. Conceptually, it is similar to behavioral PLS. Methodologically, it allowed us to test overall differences in EEG‐SpO_2_ correlations across three experimental conditions at once. We performed a mean‐centered PLS on the full 5‐min of recording, the first 150 s and the last 150 s of recordings, using nine PLS analyses in total (three EEG measures × three time intervals). In the mean‐centered PLS, the covariance matrix across participants between EEG measures and dummy variables, which coded for the experimental conditions, was decomposed into a set of LVs. Each LV was associated with: (i) a three‐dimensional vector representing the overall differences across the conditions (an overall data‐driven contrast); (ii) a *p* value from the permutation test used to test the significance of the overall contrast; and (iii) a map of frequency‐ and source‐specific z‐scores from the bootstrap test, which represented the robustness of contribution of individual EEG features to the overall contrast. Similar to behavioral PLS, we used 5000 random samples in the permutation and bootstrap tests. We then visualized the overall contrasts separately for each EEG measure, reported the corresponding *p* values, and visualized the corresponding z‐score maps. Both negative and positive z‐scores, which are large in magnitude, robustly supported the overall contrast across the conditions. Positive z‐scores directly reflected the differences across the conditions, as specified by the overall contrast. To interpret the differences across the three conditions for EEG features with negative z‐scores, we had to flip the contrast (multiplied by −1).

## 3 RESULTS

### 3.1 Participant demographics

Thirteen healthy adults (five female) with a mean age of 26.6 ± 4.0 years, mean height of 172.2 cm ± 8.8 cm, mean weight of 75 kg ± 16.3 kg, and mean BMI of 25.1 ± 3.8 kg/m^2^ successfully completed all three conditions. In total, we tested 22 participants: four participants were removed due to technical issues with our recording equipment, one due to a later disclosed psychiatric disorder, two chose to drop from the experiment after completing the first condition, one had their SpO_2_ drop below our SpO_2_ cutoff, and one had an adverse reaction to hypoxia.

### 3.2 SpO_2_ results

**FIGURE 3:**
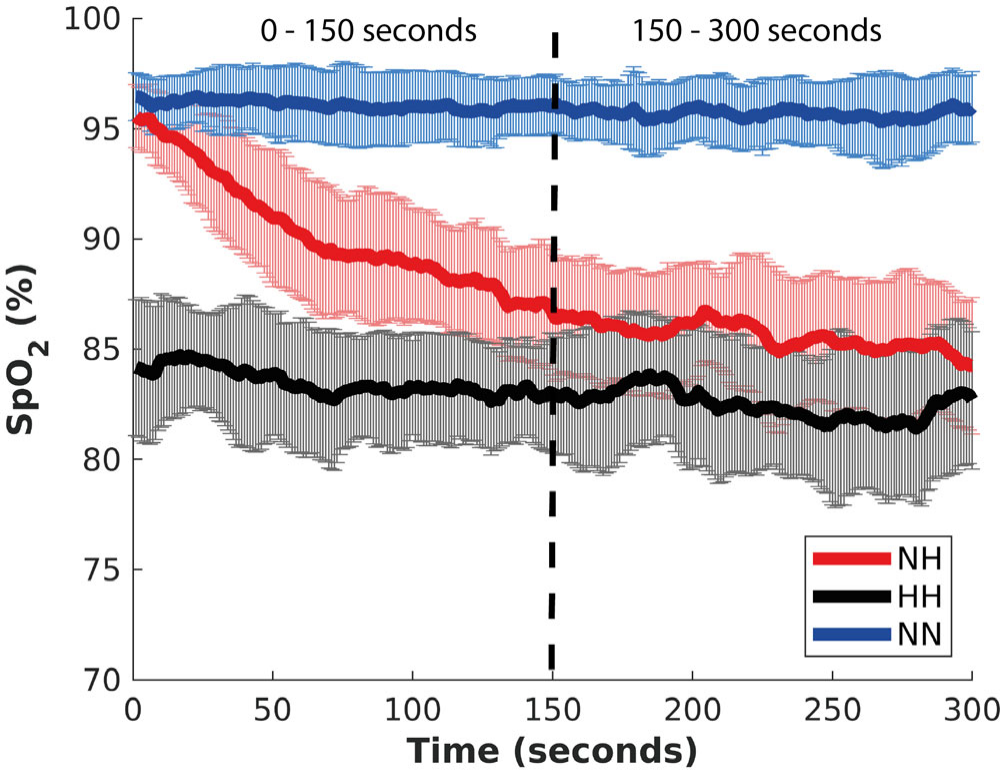
Oxygen saturation averaged over the three conditions. The three conditions were significantly different from each other, with NN having the highest SpO_2_ values, followed by NH, and HH had the lowest SpO_2_ values (*p* ≤ .0015). Data are presented as mean ± standard deviation. HH, hypobaric hypoxia; NH, normobaric hypoxia; NN, normobaric normoxia.

The mean SpO_2_ (Figure 3) over the 5‐min exposure for the NN condition was 96% ± 1.6%, 88% ± 2.7% for the NH condition, and 83% ± 3.0% for the HH condition. The SpO_2_ was significantly greater in the NN condition than both the NH (*p* < .001; FDR corrected) and HH (*p* < .001; FDR corrected) conditions; with the NH condition having significantly higher SpO_2_ than the HH condition (*p* < .001; FDR corrected). The SpO_2_ recordings began when the participants began the eyes‐open resting‐state protocol. The average decompression time was 3 min and 20 s for the HH condition, with the participants becoming hypoxic during the decompression, in comparison to the NH condition where they began to breathe the hypoxic gas mixture at the start of the protocol. Hence, the first 150 s of the recording exhibit the participants desaturating in the NH condition while in the HH condition they are already desaturated (Figure 3). Due to this desaturating occurring only in the first 150 s of the NH condition, we analyzed the last 150 s (150–300 s) of SpO_2_ once NH had entered a stable state. Analysis of the last 150 s of SpO_2_ showed that SpO_2_ was significantly greater in the NN condition than both the NH (*p* < .001; FDR corrected) and HH (*p* < .001; FDR corrected) conditions; with the NH condition having significantly higher SpO_2_ than the HH condition (*p* = .0015; FDR corrected).

### 3.3 Individual correlations obtained from behavioral PLS over 5‐min

**FIGURE 4:**
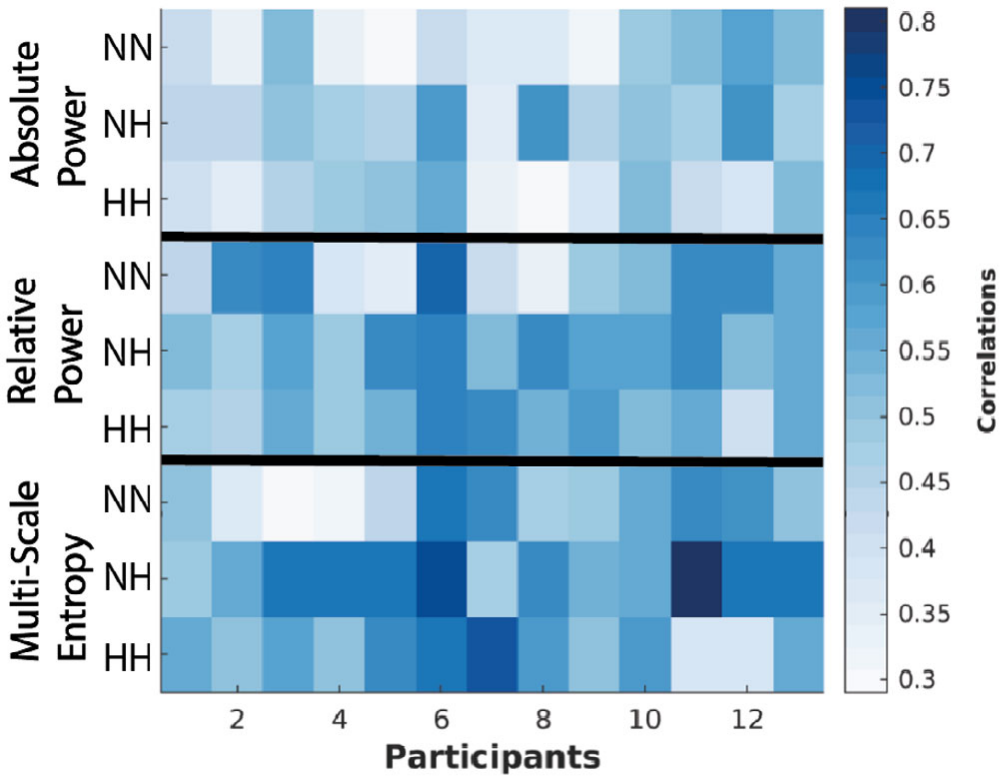
Participant‐specific overall correlations between EEG measures and SpO_2_. Each value is associated with one PLS analysis performed for one EEG measure and one participant (13 in total) in one of the three conditions. More specifically, behavioral PLS analysis was performed on each participant (i.e., 1–13) for absolute spectral power, relative spectral power, and MSE correlated with SpO_2_. The x axis stands for participants, whereas the y axis shows EEG metrics within conditions. The color bar shows the range of participant‐specific overall correlations between 0.29 and 0.81.

The individual overall correlations (across EEG epochs) from the behavioral PLS performed on each participant for brain features (absolute power, relative power, MSE) and SpO_2_ for every condition ranged from 0.29 to 0.81 (Figure 4). The results from the two tailed Wilcoxon signed‐rank test performed to compare the conditions across these overall correlations showed that NH had significantly higher correlations than NN (*p* = .0243; FDR corrected) only for MSE‐SpO_2_.

### 3.4 Spatiotemporal distribution of correlations between conditions over 5‐min

**FIGURE 5:**
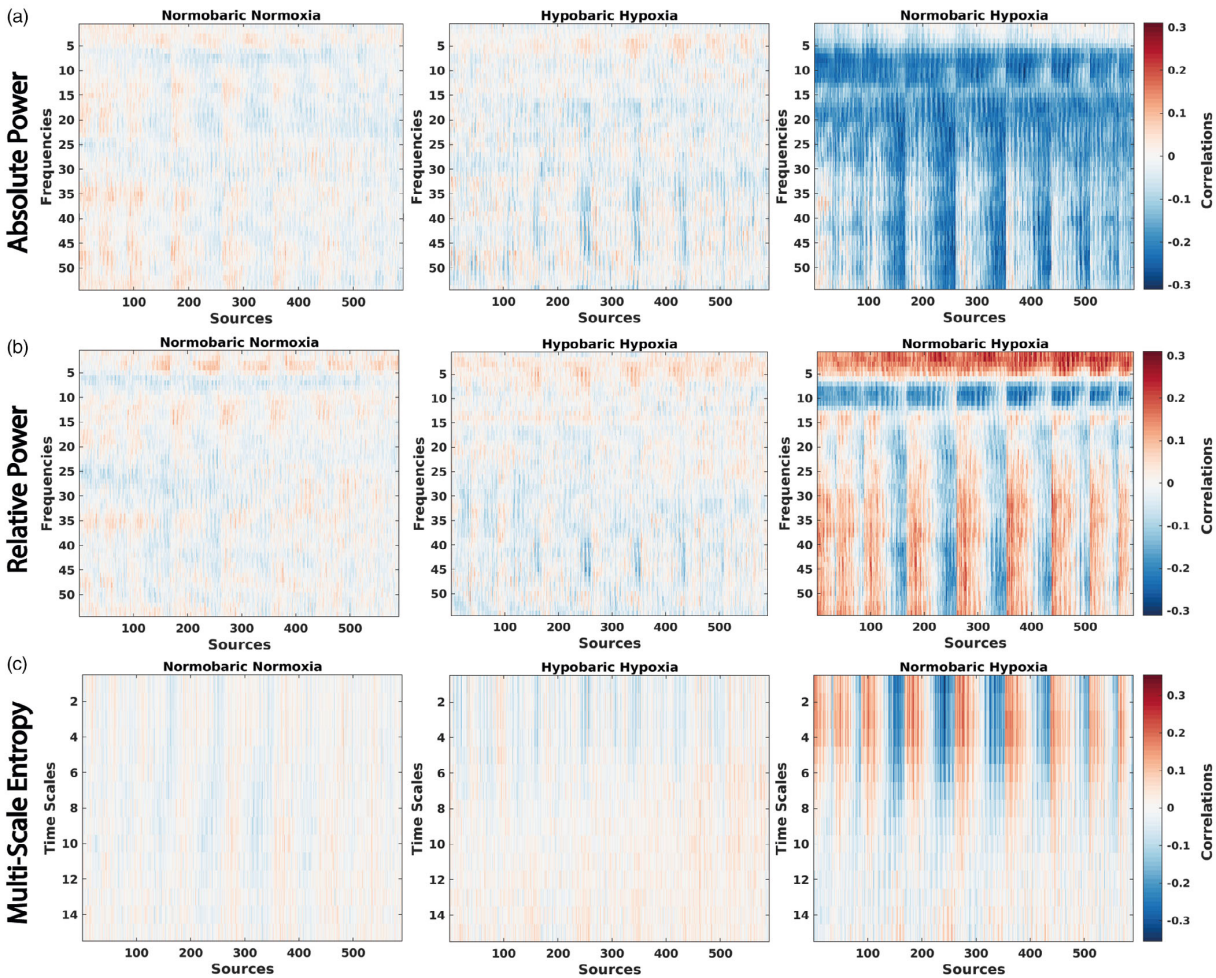
Group‐averaged correlations between EEG rhythms and oxygenation in three conditions for three EEG metrics: absolute spectral power, relative spectral power, and MSE. The correlations were averaged across participants, separately for each condition. Three subplots in one column correspond to one condition. Red values constitute a positive correlation (decrease in power with decrease in SpO_2_) and blue values represent a negative correlation (increase in power with a decrease in SpO_2_). Across the three measures, NH has stronger correlations with SpO_2_ than NN and HH when averaged across individuals. (a) Frequency source matrices for absolute power correlations with SpO_2_. The NH condition exhibits an increase in absolute power across sources with a decrease in SpO_2_. (b) Frequency source matrices for relative power correlations with SpO_2_. A prominent increase in relative power at roughly 8–12 Hz is found across sources with a decrease in SpO_2_ in the NH condition. (c) Frequency source matrices for MSE correlations with SpO_2_. The NH conditions exhibit an increase and decrease across sources in sample entropy in finer time scales with a decrease in SpO_2_.

**FIGURE 6:**
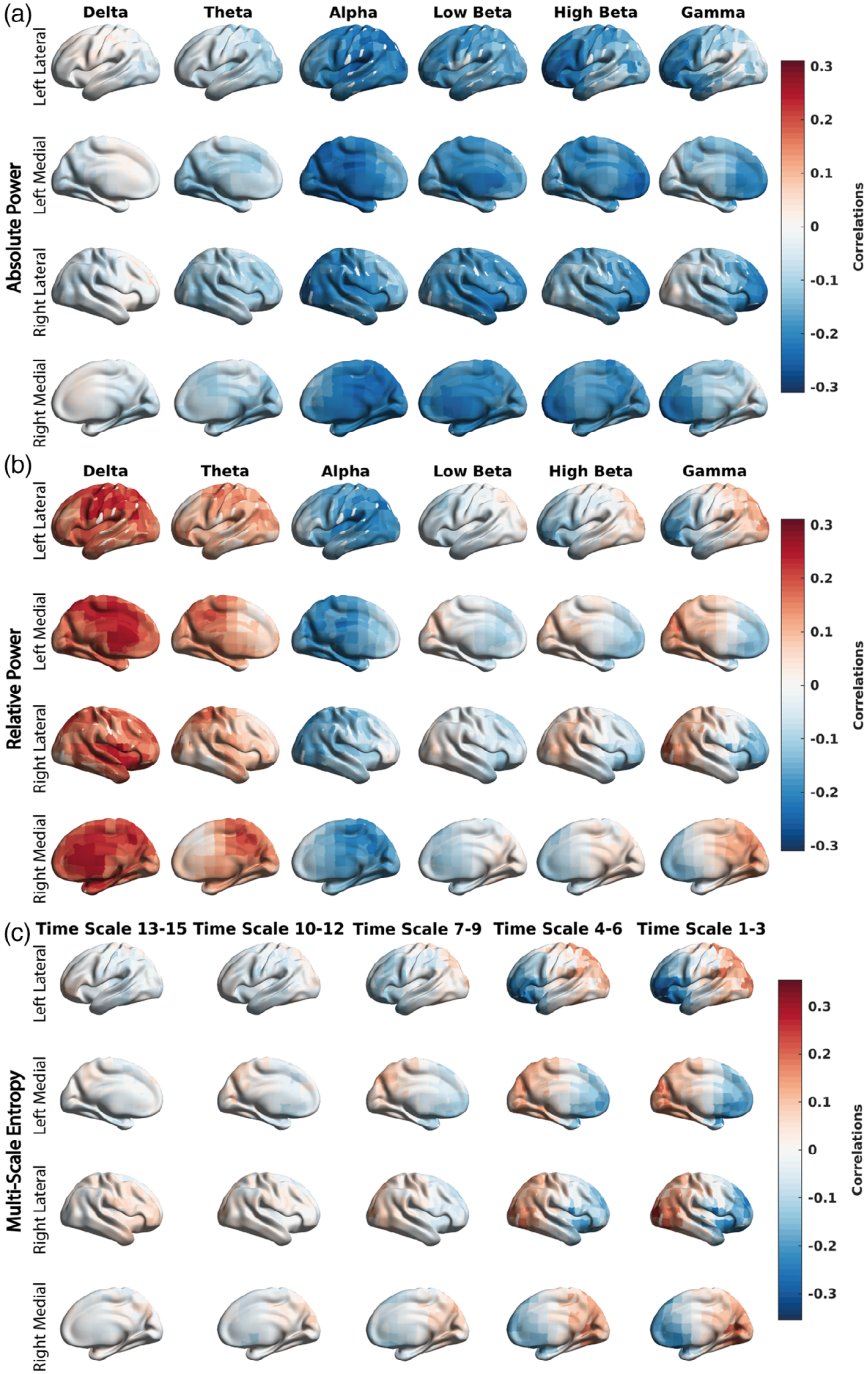
Group‐averaged correlations between EEG rhythms and SpO_2_ in NH for three EEG metrics: absolute spectral power, relative spectral power, and MSE. These are the same maps of correlations, which are shown in Figure 5 (three subplots in the right column), only this time we show them projected to the cortical surface. (a) Absolute power negatively correlated with SpO_2_ exhibits an increase in absolute power with a decrease in SpO_2_, in the alpha frequency band, alongside the low and high beta frequencies. (b) As for the relative spectral power, a negative correlation between alpha power and SpO_2_ alongside positive correlations between delta and theta power with SpO_2_ is exhibited. These correlations show a redistribution of power toward the alpha band and away from the delta and theta bands (i.e., alpha relative power increases as SpO_2_ decreases, and theta and delta relative power decrease as SpO_2_ decreases). (c) Lastly, MSE exhibits negative and positive correlations with SpO_2_, showing a dissociation of frontal and poster regions in terms of sample entropy at finer time scales (i.e., time scales 1–6), with an increase in sample entropy shown over the frontal lobe and a decrease in sample entropy shown over the posterior sections with a decrease in SpO_2_.

Given the strong individual correlations for most of the participants between EEG measures and SpO_2_ identified in the behavioral PLS analyses (Figure 4), we investigated how these overall correlations were expressed across frequency/time scales and sources, and how these spatiotemporal patterns of correlations varied across the three conditions. From each behavioral PLS analysis (for each participant), in addition to the overall participant‐specific overall correlation value between EEG and SpO_2_, we obtained a map of feature‐specific correlations (frequency‐ and source‐specific). We averaged these feature‐specific correlation maps across participants, separately for each condition and EEG measure. We found that the NH condition showed a larger spread of participant‐averaged feature‐specific correlations than the other conditions, suggesting that a difference does exist between the conditions (Figure 5). In the NH condition (Figure 6), we observed strong negative correlations between the absolute power of alpha and lower/higher beta rhythms and SpO_2_, relative to NN and HH (Figure 6). The NH condition also exhibited strong negative correlations between relative power in the alpha frequency band and SpO_2_, relative to the other two conditions. Finally, in NH, we observed strong negative correlations between sample entropy at the fine time scales and SpO_2_ in the frontal regions and strong positive correlations in the posterior regions, again relative to both NN and HH.

From a visual analysis, we noticed similar patterns in the maps of z‐scores or corresponding individual correlations for sample entropy at fine time scales (Figure 6c) and spectral power in the gamma band (Figure 6b). At the fine time scales of MSE, the new signals are close to the original signal, which also includes higher frequencies. Considering that the differences in MSE, to a large degree, can be explained by differences in spectral power (Courtiol et al., 2016; Humeau‐Heurtier, 2020), we formally correlated individual correlations (source‐specific) between EEG and SpO_2_ from two analyses: sample entropy at fine time scales and spectral power, relative and absolute, of the gamma rhythms. The correlations were evaluated across sources. Specifically, a Pearson correlation was performed on the averaged sample entropy correlations calculated at time scale 2 with average absolute power correlations at 50 Hz (*r* = .81, *p* < .001) and averaged relative power correlations at 50 Hz (*r* = .91, *p* < .001). Such strong correlations indicate a very high likelihood that sample entropy at fine time scales reflects a similar effect observed for relative spectral power for the gamma oscillations.

### 3.5 Group analysis differences in EEG–SpO_2_ correlations across conditions over the full 5‐min interval

Figure 5 shows differences in group‐averaged correlations between our EEG metrics and SpO_2_ across the three conditions. A visual analysis suggested that these differences across conditions could be robust at the group level. To formally quantify these potential differences between the conditions, we performed a mean‐centered PLS to explore differences in EEG‐SpO_2_ correlations across the three conditions at the group level. We performed the mean‐centered PLS analysis separately, for each EEG metric.

**FIGURE 7:**
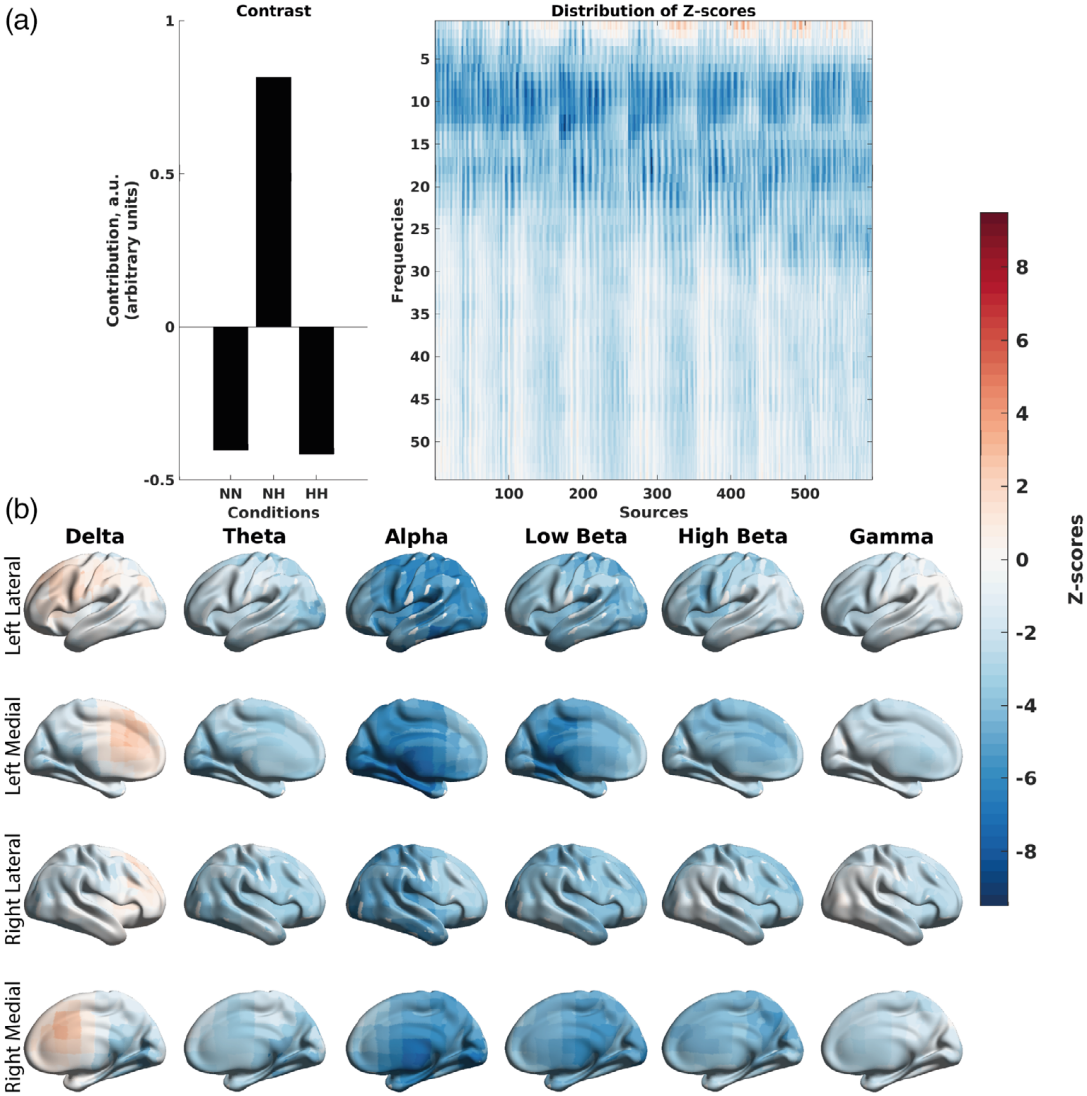
Differences in the correlations between EEG absolute power and SpO_2_ across the three conditions at the group level, as revealed by the mean‐centered PLS analysis. (a) An overall contrast across the conditions and the corresponding map of z‐scores, each associated with a unique combination of a frequency and brain region. The contrast on the left subplot differentiates NH versus NN and HH (*p* = .001). Large in magnitude negative z‐scores (shown in blue) robustly support the overall contrast, indicating lower (and in this case, more negative) correlations between EEG absolute power and SpO_2_ in the NH condition. (b) The same map of z‐scores, as in (a), but with the z‐scores shown on the brain template. The alpha rhythm exhibits the greatest effect due to hypoxia, suggesting global inhibition or a global idle brain state.

Specifically, the mean‐centered PLS performed on the absolute power correlations (Figure 7) shows that on average, these correlations were more negative in magnitude in NH, compared to NN and HH. More specifically, the vector of overall differences across conditions in Figure 7a represents a contrast of NH versus NN and HH (*p* < .001). The corresponding z‐scores were negative, on average (Figure 7b). The negative z‐scores, together with the contrast in Figure 7a, indicate that the negative correlations between the EEG power and SpO_2_ were more negative in magnitude in NH. As these correlations were on average negative (Figure 5), NH is characterized with stronger, negative correlations between the absolute EEG power and SpO_2_, compared to HH and NN. Specifically, alpha and beta absolute EEG power were negatively correlated with SpO_2_.

**FIGURE 8:**
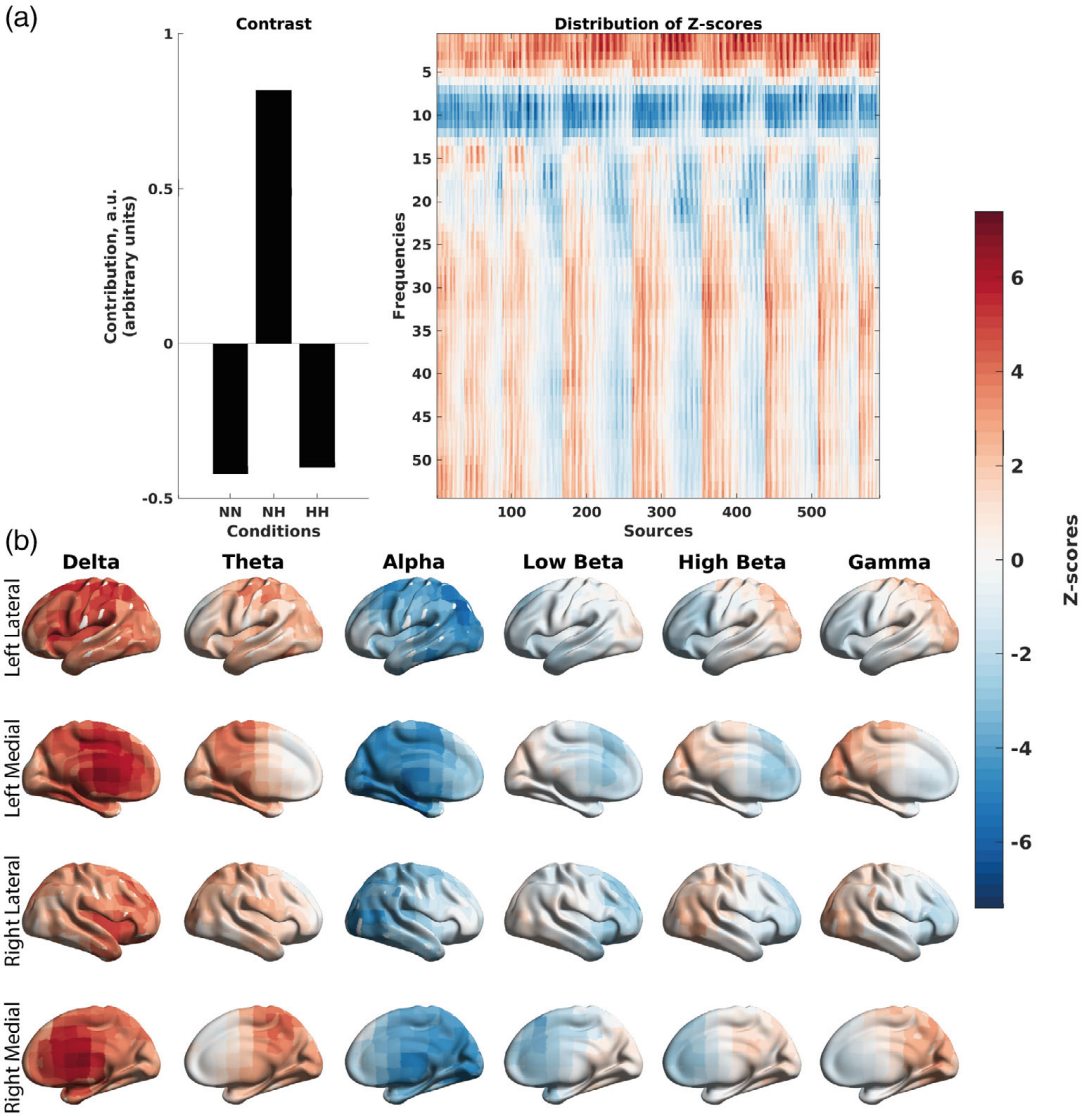
Differences in the correlations between EEG relative power and SpO_2_ across the three conditions at the group level, as revealed by the mean‐centered PLS analysis. (a) An overall contrast across the conditions and the corresponding map of z‐scores, each associated with a unique combination of a frequency and brain region. The contrast on the left subplot differentiates NH versus NN and HH (*p* = .014). Large in magnitude negative z‐scores (shown in blue) robustly support the overall contrast, indicating lower (and in this case, more negative) correlations between EEG relative power and SpO_2_ in the NH condition. Positive z‐scores (shown in red) indicate higher and more positive correlations between EEG relative power and SpO_2_. (b) The same map of z‐scores, as in (a), but with the z‐scores shown on the brain template. A shift in power is observed toward the alpha band and away from the slower frequency rhythms.

Similarly, a mean‐centered PLS performed on relative power correlations (Figure 8) showed stronger in magnitude negative correlations of relative alpha power with SpO_2_ for NH. The contrast, which was significant with *p* = .014, also exhibited NH versus NN and HH.

**FIGURE 9:**
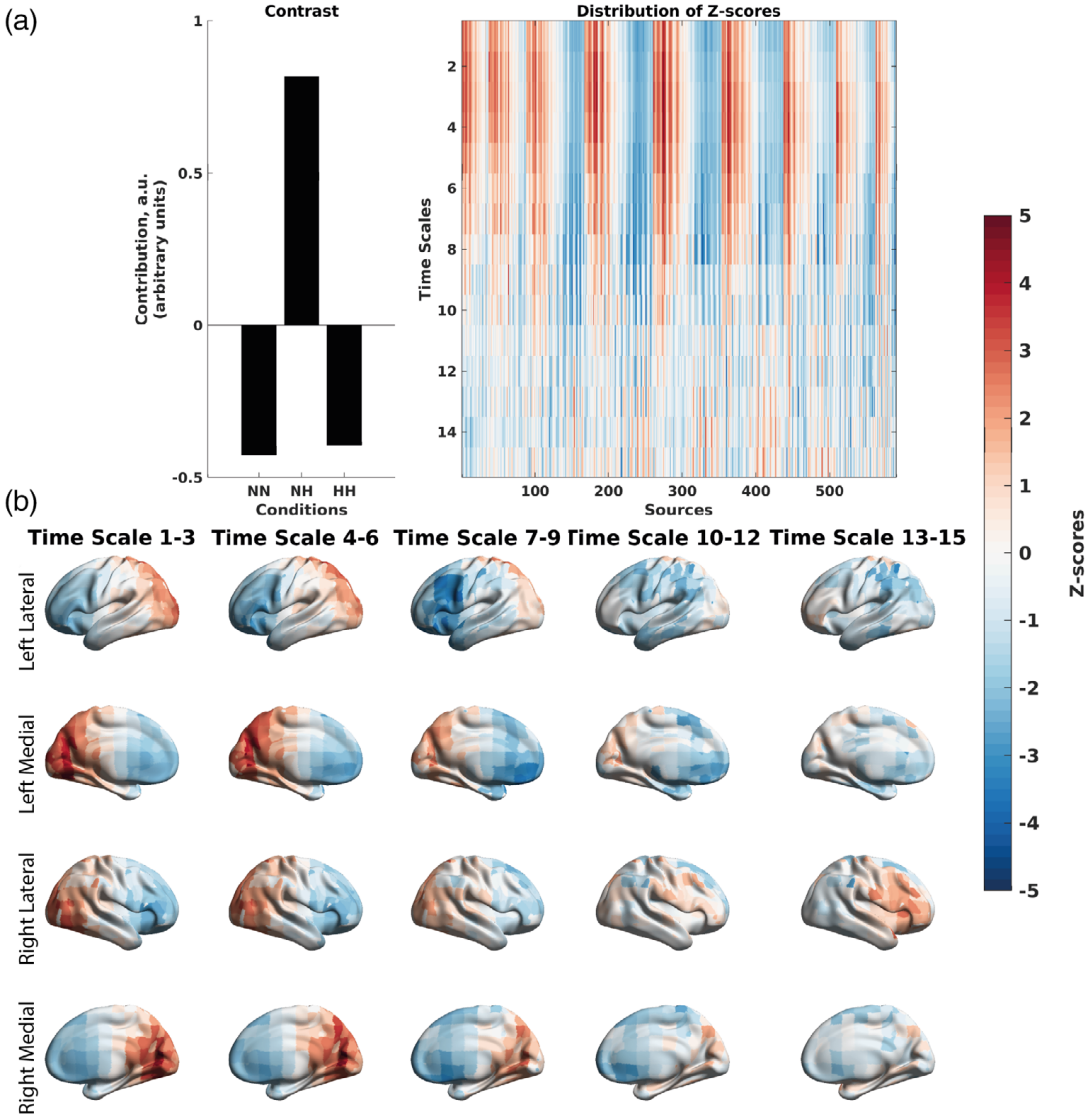
Differences in the correlations between MSE of EEG signals and SpO_2_ across the three conditions at the group level, as revealed by the mean‐centered PLS analysis. (A) An overall contrast across the conditions and the corresponding map of z‐scores, each associated with a unique combination of a frequency and brain region. The contrast on the left subplot differentiates NH versus NN and HH (*p* = .043). Large in magnitude negative z‐scores (shown in blue) robustly support the overall contrast, indicating lower (and in this case, more negative) correlations between MSE and SpO_2_ in the NH condition. Positive z‐scores (shown in red) indicate higher and more positive correlations between MSE and SpO_2_. (b) The same map of z‐scores, as in (a), but with the z‐scores shown on the brain template. The MSE results seem to show a more heterogenous spatiotemporal pattern of correlations than absolute and relative power and may be a more sensitive measure to changes related to hypoxia. At finer time scales (scales 2–6), we see an increase in sample entropy in the frontal regions (negative correlation between MSE and SpO_2_) and decrease in poster regions (positive correlation between MSE and SpO_2_). At coarser time scales (7–15), we see a reversal in the frontal cortex compared to finer time scales, with a decrease in sample entropy.

Finally, the mean‐centered PLS performed on correlations between MSE and SpO_2_ (Figure 9) showed a distinction between frontal and temporal regions at finer time scales. The frontal regions exhibited larger in magnitude negative correlations between MSE and SpO_2_. The posterior regions exhibited larger in magnitude positive correlations between MSE and SpO_2_. The contrast from the mean‐centered PLS performed on MSE correlations showed NH versus HH and NN (*p* = .043).

### 3.6 Group analysis for differences in EEG–SpO_2_ correlations across conditions for the first and second half of EEG recordings

A mean‐centered PLS for comparing correlations between EEG metrics and SpO_2_ properties across conditions was also performed on the data split in half. Specifically, the first 150 s of recording and the last 150 s of the 5‐min recording were considered separately, but compared simultaneously in PLS with six conditions (three experimental conditions times two time intervals). The rationale for such an analysis came from an observation that at the level of individual participants, the SpO_2_ level fluctuated during the entire experiment. However, on average, the first half of the 5‐min recording, when compared to the second half, was visually characterized by a higher rate of change in SpO_2_ in the NH condition (Figure 3). Exploring differences in the EEG–SpO_2_ correlations across conditions, considered separately on the first and last 150 s, but compared at the same time, allowed us to further pinpoint the nature of the robust correlations reported in Section 3.5.

Specifically, in the overall contrast from three separate mean‐centered PLS performed to compare the three conditions and two time intervals at the same time revealed that the first 150 s of the NH condition significantly differed from the other conditions. These results suggested that we could be more specific with the correlation patterns reported in Section 3.5: the desaturation occurring during the first 150 s of the NH condition drove the significant differences across the conditions, when considered for the entire 5 min of the experiments.

**FIGURE 10:**
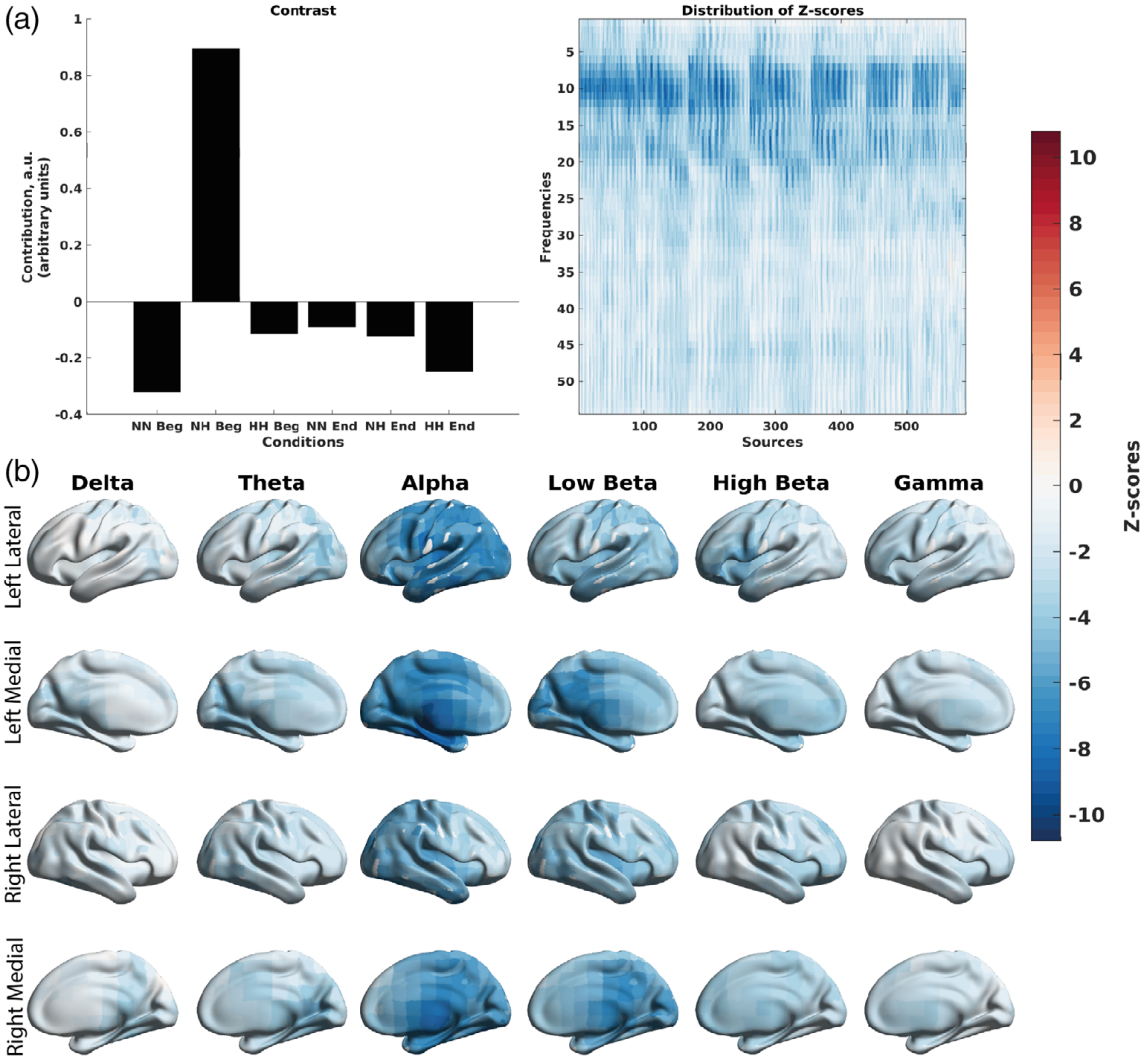
Differences in the correlations between EEG absolute power and SpO_2_ across the three conditions at the group level split into the first and last 150 s of the recording, as revealed by the mean‐centered PLS analysis. (a) An overall contrast across the conditions and the corresponding map of z‐scores, each associated with a unique combination of a frequency and brain region. The contrast on the left subplot differentiates the first 150 s of NH versus the last 150 s of NH and the first and last 150 s of NN and HH (*p* < .001). Large in magnitude negative z‐scores (shown in blue) robustly support the overall contrast, indicating lower (and in this case, more negative) correlations between EEG absolute power and SpO_2_ in the NH condition. (b) The same map of z‐scores, as in (a), but with the z‐scores shown on the brain template. The alpha rhythm exhibits the greatest effect due the first 150 s of the NH condition. This suggests that the global inhibition or a global idle brain state due to the alpha rhythm is due to the active desaturation occurring during the first 150 s of NH. HH Beg, first 150 s of hypobaric hypoxia; HH End, last 150 s of hypobaric hypoxia, NH Beg, first 150 s of normobaric hypoxia; NN Beg, first 150 s of normobaric normoxia; NH End, last 150 s of normobaric hypoxia; NN End, last 150 s of normbaric normoxia.

Specifically, the mean‐centered PLS based on the absolute power correlations (Figure 10) showed that through the contrast (Figure 10a) on average these correlations were more negative in magnitude during the first 150 s of the NH condition, versus the last 150 s of NH condition, and the first and last 150 s of the HH and NN conditions (*p* < .001). More specifically, the first 150 s of the NH condition exhibited stronger negative correlations between alpha and low beta power and SpO_2_, which were spread diffusely over the cortex (Figure 10b).

**FIGURE 11:**
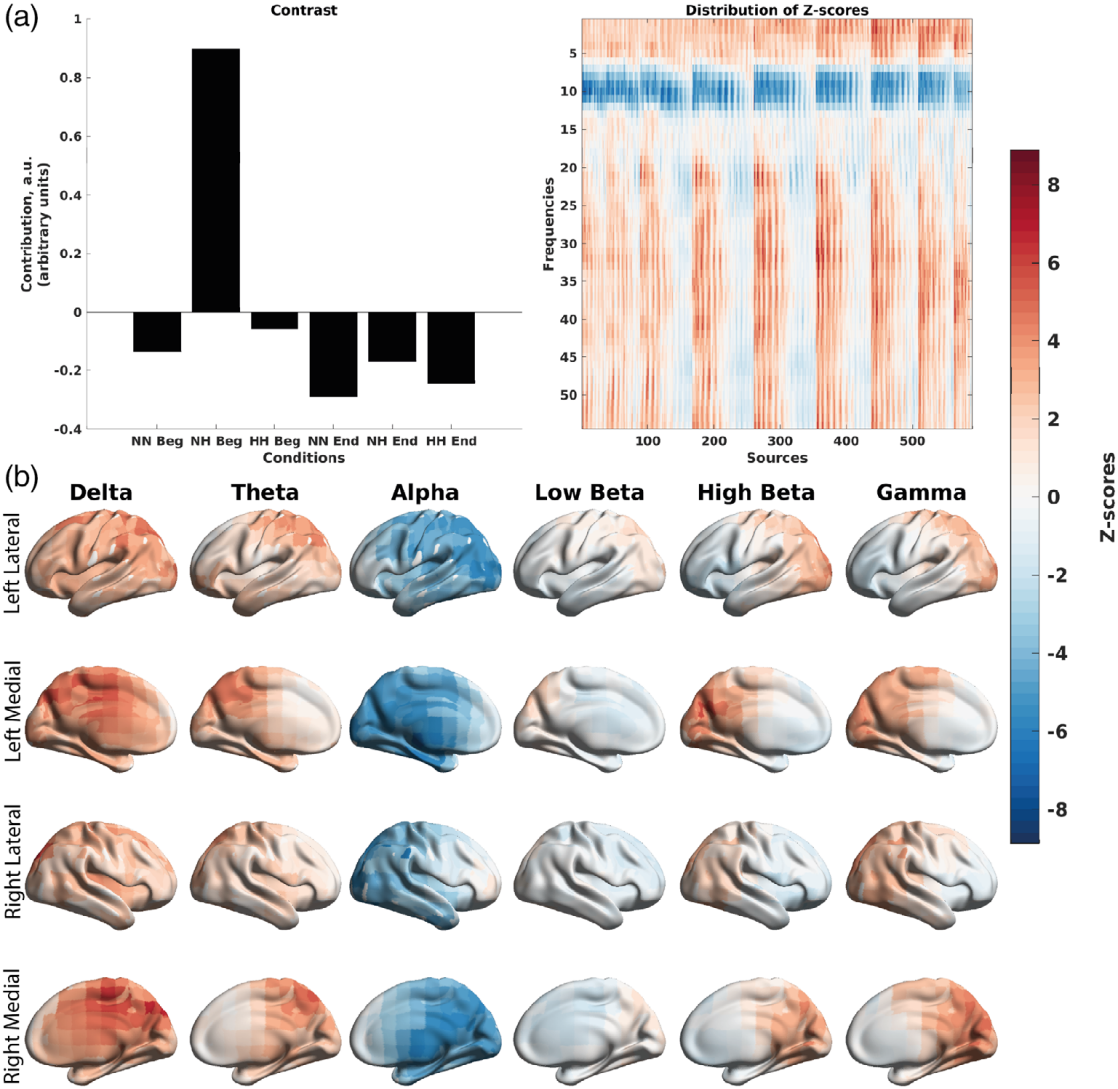
Differences in the correlations between EEG relative power and SpO_2_ across the three conditions at the group level split into the first and last 150 s of the recording, as revealed by the mean‐centered PLS analysis. (a) An overall contrast across the conditions and the corresponding map of z‐scores, each associated with a unique combination of a frequency and brain region. The contrast on the left subplot differentiates the first 150 s of NH versus the last 150 s of NH and the first and last 150 s of NN and HH (*p* = .001). Large in magnitude negative z‐scores (shown in blue) robustly support the overall contrast, indicating lower (and in this case, more negative) correlations between EEG relative power and SpO_2_ in the NH condition. Positive z‐scores (shown in red) indicate higher and more positive correlations between EEG relative power and SpO_2_. (b) The same map of z‐scores, as in (a), but with the z‐scores shown on the brain template. A shift in power is observed toward the alpha band and away from the slower frequency rhythms. This shift in power is observed during the active desaturation phase of the first 150 s of NH. HH Beg, first 150 s of hypobaric hypoxia; HH End, last 150 s of hypobaric hypoxia; NH Beg, first 150 s of normobaric hypoxia; NN Beg, first 150 s of normobaric normoxia; NH End, last 150 s of normobaric hypoxia; NN End, last 150 s of normbaric normoxia.

Similarly, a mean‐centered PLS performed on relative power correlations (Figure 11) showed stronger in magnitude negative correlations between relative alpha power and SpO_2_, and positive correlations between delta, theta, high beta and gamma power and SpO_2_ for the first 150 s of NH. The significant contrast (*p* = .001) in Figure 11a exhibited the pattern of the first 150 s of the NH condition versus the last 150 s of the NH condition, and the first and last 150 s of the HH and NN conditions. Specifically, the first 150 s of the NH condition showed strong negative correlations for relative alpha power and SpO_2_, spread diffusely over the cortex, with the positive correlations between gamma, high beta, and delta relative power and SpO_2_ spread in the posterior half of the cortex, and the positive correlations between delta relative power and SpO_2_ spread diffusely over the cortex (Figure 11b).

**FIGURE 12:**
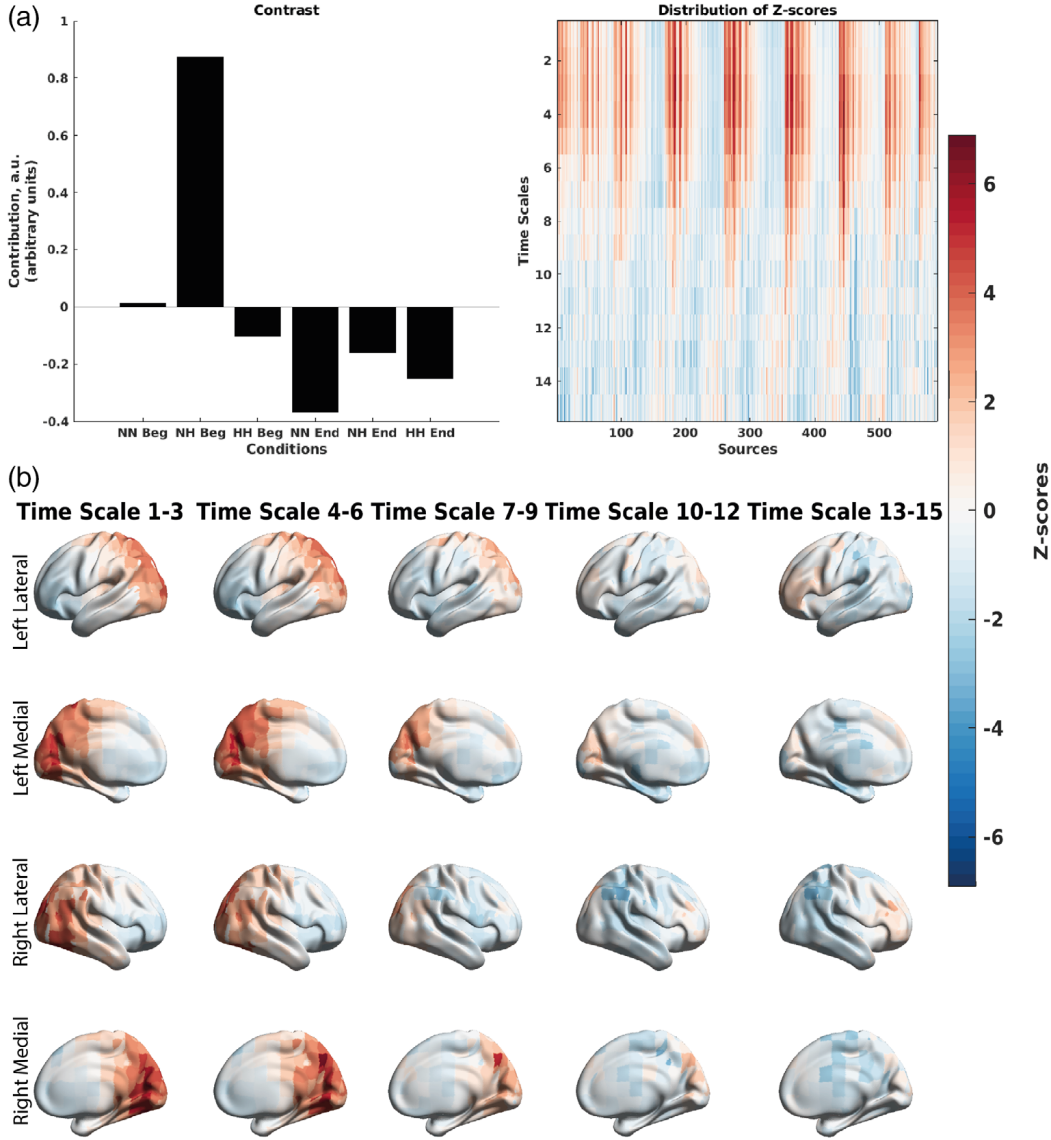
Differences in the correlations between MSE and SpO_2_ across the three conditions at the group level split into the first and last 150 s of the recording, as revealed by the mean‐centered PLS analysis. (a) An overall contrast across the conditions and the corresponding map of z‐scores, each associated with a unique combination of a frequency and brain region. The contrast on the left subplot differentiates the first 150 s of NH and the negligible first 150 s of NN versus the last 150 s of NH and NN, and the first and last 150 s of HH (*p* = .014). Large in magnitude positive z‐scores (shown in red) robustly support the overall contrast, indicating higher (and in this case, more positive) correlations between MSE and SpO_2_ in the first 150 s of the NH condition. Negative z‐scores (shown in blue) indicate lower and more negative correlations between MSE and SpO_2_. (b) The same map of z‐scores, as in (a), but with the z‐scores shown on the brain template. The MSE results seem to show a distinct spatiotemporal pattern of correlations, with a clear distinction of the posterior section of the cortex exhibiting positive correlations occurring during the first 150 s of the NH condition. At finer time scales (scales 2–6), we see a decrease in sample entropy in poster regions (positive correlation between MSE and SpO_2_). HH Beg, first 150 s of hypobaric hypoxia; HH End, last 150 s of hypobaric hypoxia; NH Beg, first 150 s of normobaric hypoxia; NN Beg, first 150 s of normobaric normoxia, NH End, last 150 s of normobaric hypoxia; NN End, last 150 s of normbaric normoxia.

Finally, the mean‐centered PLS performed on MSE–SpO_2_ correlations (Figure 12), which showed stronger in magnitude positive correlations between sample entropy at time scales 1–6 (fine time scales) and SpO_2_ (*p* = .014). The contrast (Figure 12a) can be interpreted as the first 150 s of the NH condition and the neglible first 150 s of NN versus the last 150 s of the NH condition, last 150 s of the NN condition, and the first and last 150 s of the HH condition. The positive correlations between MSE at the fine time scales and SpO_2_ were spread over the posterior half of the cortex (Figure 12b).

### 3.7 Mean‐centered PLS analysis to test for conditional differences in the last half of data

We performed another mean‐centered PLS on solely the last 150 s of data to further elucidate our previous findings that no conditional differences exist in the last 150 s of the data, as the first 150 s of the NH recording may have masked other differences. The correlations between absolute power and SpO_2_ were not significant (*p* = .668), as were the correlations between relative power and SpO_2_ (*p* = .851), and the correlations between MSE and SpO_2_ (*p* = .748). This confirms the previous mean‐centered PLS results that no difference exists between our conditions at our hypoxic dose.

## 4 DISCUSSION

We demonstrated significant differences in correlations between SpO_2_ and EEG oscillations (absolute power, relative power, and MSE) during NH compared to HH and NN. These differences were not due to the hypoxic condition per se, but rather reflected the onset of the hypoxic stimulus during NH. Unfortunately, we did not record the HH condition during desaturation and could only compare desaturating NH to already desaturated HH. Based on our results, we showed that the impact of desaturation on neurophysiological function during NH followed a distinct spatiotemporal pattern of correlations from the already desaturated HH condition. The HH and NH conditions achieved a similarly low final SpO_2_ near the end of the 5‐min, with the HH condition achieving a lower *average* SpO_2_ for the course of the experiment which produced neurophysiological responses that were in fact similar to the NN conditions. The last half of the recording, when the NH condition had desaturated, showed that the NH, HH, and NN conditions did not differ, suggesting that for the chosen level of hypoxia barometric pressure (HH) did not result in any distinct neurophysiological differences.

The difference in the oxygen stimulus during the first 150 s of the recording occurred because our participants breathed ambient air in the hypobaric chamber during decompression to the simulated altitude of 3962 m in the HH condition. The implication is that the participants were starting to become hypoxic during the decompression time, and their SpO_2_ had already hit a plateau (desaturated) before the experiment started. Conversely, in the NH condition the participants were switched from ambient to hypoxic air at the start of the experiment, so the first part of the data recording occurred while their SpO_2_ was still decreasing (desaturating). This difference in oxygen stimulus is why we decided to split our data in half, in order to better elucidate whether our results were due to the hypoxic condition or desaturation occurring in the NH condition. Future research is needed to further investigate whether the brain follows a specific spatiotemporal pattern during desaturation as suggested by our results, and whether this desaturation pattern differs between NH and HH.

We chose to perform correlations at the individual level between brain measures and SpO_2_ due to the individual variability in hypoxia tolerance. We used this individual variability, shown by the variance in SpO_2_ values in our study (Figure 3), to probe the spatiotemporal pattern of the correlations between our brain measures and SpO_2_. Using this approach, we showed that the desaturation phase of the NH condition is highly homogenous across participants in regards to the spatiotemporal pattern of brain activity correlated with SpO_2_. The larger spread of correlations implies that there was less individual variability in how the brain responded to the desaturation phase (0–150 s) of the NH condition compared to the NH and HH conditions where the SpO_2_ was relatively stable. The desaturation phase of the NH condition exhibited an increase in alpha power correlated with decreased SpO_2_ which is in line with previous research finding an increase in alpha power during eyes‐open states (Papadelis et al., 2007; Schellart & Reits, 2001). The first 150 s of the NH condition also exhibited a large redistribution of energy across time and space as shown by the large negative alpha relative power correlations and positive delta and theta power correlations with SpO_2_, and the distinct posterior spatial distribution of positive correlations of sample entropy at finer time scales with SpO_2_. Future research is needed to further investigate whether the brain follows a specific spatiotemporal pattern during desaturation as suggested by our results, and whether this pattern differs between NH and HH.

Hypoxia depletes energy substrates and therefore alters transmembrane potentials, and this may explain the pattern of spatiotemporal correlations found during the first 150 s of the NH condition. The brain requires a balance between energy substrate supply and energy substrate demand. Oxygen is vital for aerobic respiration by the mitochondria in order to create ATP, which is then used to maintain the sodium‐potassium pump (Na^+^/K^+^ATPase). The Na^+^/K^+^ATPase maintains resting membrane potential by transporting two potassium ions into the cell and three sodium ions out for the cost of an ATP molecule (Martin et al., 1994). During hypoxia, this system is disrupted, as the brain will continue to use glucose but will not be able to produce adequate levels of ATP. Due to insufficient ATP levels, the Na^+^/K^+^ATPase will not function properly, as ATP is required by the Na^+^/K^+^ATPase to maintain resting membrane potential (Somjen, 2001). The inability of the cell membrane to maintain its resting membrane potential in turn results in widespread dysregulation of synaptic transmissions and potential functional isolation of neuronal cell populations (Martin et al., 1994; Papadelis et al., 2007).

The widespread cell membrane dysregulation will have a detrimental impact on neuronal oscillations, which is the fluctuation of the postsynaptic potentials. Neurons communicate with each other through the release of neurotransmitters, with GABA being the main inhibitory transmitter and glutamate the main excitatory transmitter, that travel and bind to receptors on the receiving neuron. These neurotransmitters form the basis of neuronal oscillations, as the postsynaptic potential will fluctuate between periods of excitability and inhibition; therefore, the oscillation contains a temporal period of peak excitability (Fries, 2005). A neuronal population sending an output needs to time this output so that it arrives during the peak excitability of the receiving neuronal population. Hypoxia induced widespread membrane dysregulation will therefore have a detrimental effect on neuronal oscillations as the neuronal cell membrane will not be able to fluctuate normally between periods of excitability and inhibition. Large groups of neuronal populations can synchronize their firing patterns, which leads to oscillations at different frequencies, which is expressed as the main EEG frequency bands. Thus, the detrimental effect of hypoxia on neuronal oscillations will subsequently impact frequency band power.

Previous research also found that the alpha and beta frequency bands are sensitive to lower levels of hypoxia. Ozaki et al. (1995) found that the spectral power of alpha started to decrease at 3000 m and was almost absent at 6000 m. This highlights the sensitivity of the alpha frequency band to hypoxia, as it decreased even at the “mild” hypoxic stimulus of 3000 m. Our study supports these findings as Ozaki et al. (1995) utilized an eyes‐closed protocol resulting in a decrease in alpha power, while our study utilized an eyes‐open protocol resulting in our negative alpha power correlation (Schellart & Reits, 2001). Schneider and Strüder (2009) observed an increase in low beta (12.5–18 Hz) power in the right superior frontal gyrus with a hypoxic dose of 95.26 mmHg of oxygen, similar to our hypoxic stimulus. Alpha power, and potentially low beta power, may be the dominant frequency bands for lower hypoxic levels.

Similar changes in alpha and beta power are observed with the anesthetic propofol, which like severe hypoxia, can lead to the loss of consciousness. During the gradual administration of propofol, an increase in beta and gamma power appears at the early stages of sedation with increased doses leading to a shift in power toward alpha (Ching et al., 2010). Higher doses of propofol lead to a loss of consciousness that is accompanied by a large increase in alpha power (Purdon et al., 2013). Given that loss of consciousness can occur with hypoxia, our negative correlations of alpha and beta absolute power with SpO_2_, alongside the negative correlation of alpha relative power and SpO_2_, may reflect a similar mechanism to propofol. Furthermore, Schneider and Strüder (2009) found a similar increase in beta power at a low hypoxic level similar to our own, mirroring the beta power increase in low‐dose propofol administration. This suggests that mild hypoxia may exhibit similar spectral power changes in the alpha and beta bands as a mild anesthetic.

Alpha band activity is thought to play an inhibitory role that may be utilized to conserve oxygen during hypoxia. In particular, alpha oscillations are inhibitory resulting in a cyclic inhibition that modulates the time window for sensory processing (Jensen & Mazaheri, 2010). When alpha power is high, it reflects high inhibition resulting in a shorter time window for sensory processing (Jensen & Mazaheri, 2010). Given that hypoxia may disrupt normal neuronal oscillations by causing widespread cell membrane dysregulation, this disruption could lead to the cortex wide negative correlation of alpha power with SpO_2_ signifying alpha inhibition during the desaturation phase of the NH condition. Furthermore, previous studies have found a negative correlation between alpha power and the BOLD response, further suggesting that alpha inhibition may reflect idling of the cortex due to decreased neural activity (Chawla et al., 1999; Mayhew et al., 2013; Pang & Robinson, 2018; Wenzel et al., 2000). Speculatively, this widespread inhibition reduces the processing capabilities of the brain, which can be a protective measure to preserve ATP during hypoxia. During the desaturation phase of hypoxia, the brain must either regulate oxygen expenditure in an unstable environment until the level of oxygen has stabilized or enter an idle state to conserve oxygen. Once the desaturation phase has plateaued, the brain enters a steady state again where it would either decrease the inhibitory alpha regulation of metabolism or leave the idle state. Since our hypoxic dose could be considered mild in relation to other studies, our negative correlation of alpha absolute and relative power with SpO_2_ may represent a protective measure to conserve ATP as the brain starts desaturating. Whether this is an innate regulatory action to conserve ATP, or simply an idle brain state that utilizes less ATP is not known.

Further evidence of the brain entering a lower metabolic state during hypoxia was shown by Rozhkov et al. (2009), who found that after 20 min of breathing an 8% oxygen mixture participants had a slowing and interelectrode synchronization of EEG signals during an eyes‐open resting‐state task. The authors postulated that this meant the hypoxic subjects had entered a lower functional state, and that this lower functional state would be unsuited for normal cognitive functions. However, this lower functional state may be adaptive for hypoxia, as it means that neurons require less energy (i.e., less oxygen), enabling sufficient energy is available for essential activities necessary to survive hypoxia. They also found a rearrangement of electrical activity to more lateral regions, suggesting involvement of the temporal lobes and therefore activation of the limbic system. The limbic system is used in controlling autonomic function, and so it appears that the hypoxic brain channels the necessary resources to brain areas necessary for survival.

The addition of MSE to our more traditional spectral power analysis has provided novel insight over and above the traditional metrics, allowing us to better investigate the hypoxic brain. MSE falls under the framework of nonlinear dynamics and is interpreted as the rate of information generated by a dynamic system underlying the observed signal (Richman & Moorman, 2000). Using MSE, we observe the most robust effects (large in magnitude z‐scores, which show the robustness of contribution of individual features) at fine time scales (Figure 5c). Taking into account that coarse time scales are equivalent to low‐pass filtering, our most robust effect for MSE in the fine time scales is based on a wide frequency range, which includes gamma oscillations (Courtiol et al., 2016). Furthermore, we found a strong correlation between z‐scores at the fine time scale 2 and z‐scores for relative and absolute spectral power at 50 Hz (gamma rhythm). Spectral power and MSE can be collinear, due to their shared linear signal characteristics (e.g., rhythmicity and temporal autocorrelations) with previous work finding that differences in power can account for MSE effects (Kosciessa et al., 2020). Thus, it seems that MSE emphasizes an effect supported by the gamma rhythms. This is further emphasized by the similarity in the spatial maps of z‐scores between gamma relative power (Figure 6c) and time scale 1–3 (Figure 6b). The corresponding spatial maps of z‐score show a redistribution of signal complexity across the frontal and posterior regions (Figure 6c). In turn, changes in signal complexity are associated with decreases or increases in brain connectivity (Vakorin, 2011). On the other hand, gamma rhythms are known to support the binding of parallel information processing, contributing to cognition and formation of short‐term memory. Hypoxia can block gamma activity, for example, in the hippocampus (Villasana‐Salazar et al., 2020), which can be particularly susceptible to hypoxic injury (Zhang et al., 2022). In turn, interactions between the hippocampus and frontal areas are important for memory processes (Preston & Eichenbaum, 2013). Also, the hippocampus interacts with the posterior regions in memory retrieval (Ciaramelli et al., 2020). Thus, speculatively, our MSE results indicate a detrimental effect of abrupt changes in oxygenation as expressed by the NH condition (in particular, the first 150 s of recording) in hypoxia on memory and potentially cognition.

The sensitivity of MSE to hypoxia makes it a good candidate for potential wearable flight monitoring devices of hypoxia, as an EEG sensor may be placed in a helmet to monitor for inflight hypoxic biomarkers. Indeed, a real‐time sensor has been developed to identify hypoxia biomarkers in the exhaled air of military pilots, attesting to the interest in this avenue of research (Harshman et al., 2015). A similar hypoxia sensor could also be developed for occupational and technical divers, as they also run the risk of experiencing hypoxia; with a recent study advocating for the development of pulse oximetry for underwater use to warn divers of hypoxia (Lance et al., 2022). Future research into these biomarkers is needed, but potential hypoxia biomarkers may be the finer time scale decrease in MSE alongside concurrent increases in alpha activity, because this is the frequency band that reports most faithfully on the neurophysiological responses to hypoxia. Both the finer time scales and alpha activity were significantly correlated with the first 150 s of the NH condition during which participants were desaturating, which is the most critical time for a sensor to warn of hypoxia. Depending on the level of the hypoxic stimulus, the desaturation phase of hypoxia in a real‐world scenario is the critical time during which one must notice that they are becoming hypoxic and work to solve the issue of hypoxia before they become too hypoxic to function.

## 5 LIMITATIONS

Participants in the NH condition started the recording with their SpO_2_ at normal levels and then desaturated and stabilized, while in the HH condition participants were already desaturated and their SpO_2_ had stabilized. This is due to participants breathing ambient air in the hypobaric chamber while we depressurized the chamber to 3962 m, meaning they were slowly becoming hypoxic during the depressurization process. Conversely, in the NH condition, participants were switched to the hypoxic gas mixture at the start of the experiment, which led to them desaturating and having their SpO_2_ levels drop during the recording. Due to difference in the timing of onset of hypoxia, we did find that the desaturating segment of NH had a distinct and significantly different spatiotemporal pattern of correlations when compared to the already desaturated HH condition and desaturated segment of the NH condition. With this in mind, the desaturation phase of the NH condition may not be a true limitation in the study, as it represents the real‐world scenario of a sudden loss of airplane cabin pressure and subsequent desaturation during which a pilot must quickly mitigate this sudden desaturation. We were not able to record while the HH condition desaturated, and therefore we cannot state whether the spatiotemporal pattern of desaturation during NH was similar to HH desaturation. In terms of our original research question of whether a difference exists between NH and HH, a future publication will better address this issue, as it will compare responses during NH and HH once the SpO_2_ had stabilized.

## 6 CONCLUSION

The current literature is lacking in studies directly comparing how hypoxia effects brain activity in NH and HH. Our results add to this picture, while also raising novel areas of future investigation. We demonstrated the utility of MSE in the evaluation of neurophysiological responses to hypoxia, in addition to traditional EEG metrics. We showed that the desaturation phase of NH exhibited a robust spatiotemporal pattern of brain activity alongside a large redistribution of energy in the brain, and then once a stable state of SpO_2_ was reached the NH and HH conditions did not differ. Our results suggest that neurophysiological responses to NH and HH, when initiated using common laboratory paradigms, do not differ between NH and HH at our hypoxic dose. Importantly, we found that the brain responds differently to changing SpO_2_ than to steady‐state hypoxia. One application of our findings is that HAI training courses should focus on the desaturating phase of hypoxia, as this has a robust pattern of brain activity and is the critical period during which one must mitigate the hypoxic stimulus. Future study is needed to further elucidate these findings to determine whether the increase in alpha correlated with SpO_2_ decrease is an innate regulation of metabolic activity.

## CONFLICT OF INTEREST

The authors have no conflict of interests to declare.

## Data Availability

The data that support the findings of this study are available from the corresponding author upon reasonable request.
